# Are tetraplegic handbikers going to disappear from team relay in para-cycling?

**DOI:** 10.3389/fphys.2013.00077

**Published:** 2013-04-09

**Authors:** Thierry Weissland, Pierre-Marie Leprêtre

**Affiliations:** Laboratoire de Recherche Adaptations Physiologiques à l'Exercice et Réadaptation à l'Effort, EA-3300, UFR-STAPS, Université de Picardie Jules VerneAmiens, France

Since 2007, the International Cycling Union (UCI) and International Paralympic Committee (IPC) organize the most prestigious competitions bound for the athletes with locomotor and sensorial impairments: the Paracycling World Championships, the Para-cycling World Cup and the Paralympic Games (http://www.uci.ch/Modules/BUILTIN/getObject.asp?MenuId=andObjTypeCode=FILEandtype=FILEandid=MzIyNjAandLangId=1, p 3). Based on the functional disability, male and female athletes are divided into four groups of disability for all UCI age categories. One of four, the hand-cycling, includes Women (WH) and Men (MH) whom ride on hand tricycles in prone (i.e., arm power) or kneeling (i.e., arm-trunk power) positions. Based on the anatomic level of spinal cord injury and associated functional limitations (or similar), hand-cycling category contains four classes (H1, H2, H3, and H4) from the most (H1: impaired sympathetic nerve system) to the less (H4: arm cranking ability in kneeling position) limited ability (UCI Rules. Cycling Regulation.– E0712. Part XVI: Para-cycling; Chapter V-16.5.002–16.05.005, 2012). To promote hand-cycling category, UCI inaugurated during the road world championship in 2009 (Bogogno, Italia) a new model of competition, the team relay (TR), then introduced it in road world cup and recently in the Paralympic Games of London 2012.

For all para-cycling TR competitions, a team shall be composed of three mixed athletes, including an athlete with a scoring value of one point, for a maximal total of six points per team. Hence, males with completed cervical spinal injury (C6–C8) are worth one point (G1: MH1), two and three points for their counterparts with completed spinal cord injury from 1st to 10th thoracic vertebrae (G2: MH2) and from 11th thoracic vertebrae or below (G3: MH3 and G4: MH4), respectively. Based on the time trial performance (TT) realized during the last Paralympic games, our chronometric analysis showed, in fact, a decrease in TT according to athlete scoring value and so athlete's impairment (Table 1). The average of the 5 best times to perform 16 km TT was significant higher for G1 (35 min 55 s ± 2 min 26 s) compared to G2 (31 min 10 s ± 3 min 35 s) and G3 (26 min 14 s ± 0 min 57 s) (*p* < 0.05). Surprising, female athletes are only included in two categories: WH1 and WH2 are credited of one point and WH3 and WH4, two points (UCI Rules. Cycling Regulation.– E0712. Part XVI: Para-cycling; Chapter V, 16.5.012, 2012). If this scoring system invites to insert women into the TR for success, it raises the question about the place of the most limited athletes in a successful team. In H1 class, male and female athletes present an impaired sympathic nerve system due to a tetraplegia or similar functional ability profile although MH2 and WH2 are paraplegic or equivalent. The MH1 -WH2 mixing supposes to consider a similar cycling performance between tetraplegic male and paraplegic female. However, our chronometric lap time analysis during the last international competitions of Roma, Segovia, and London showed a potential gender-disability effect among athletes with the same scoring value. For example in Table 1, averaged lap time was significantly lower of 31.0 ± 9.5 s for WH2 winner compared to her MH1 counterpart during these three competitions.

**Table 1:** **Time (in min:sec ± SD) of the 5 first final time trial (16 km) for division H1–4 men and women during the paralympic games in brands Hatch, the 5 September 2012**.

	**1 point**		**2 points**			**3 points**	
	**MH1 min:sec**	**WH2 min:sec**	**MH2 min:sec**	**WH3 min:sec**	**WH4 min:sec**	**MH3 min:sec**	**MH4 min:sec**
Time TT	37:24 ± 1:31	34:25 ± 2:20	27:16 ± 0:29^*^	34:46 ± 1:28	31:28 ± 2:44^*^	26:44 ± 0:56^*^^δ^^ϒ^	25:44 ± 0:44^*^^δ^^ϒ^
(Min–Max)	(35:41–39:03)	(31:06–37:14)	(26:52–28:02)	(33:21–36:39)	(28:18–34:26)	(25:24–27:52)	(24:50–26:36)
RO Lap	5:37 ± 0:19	5:00 ± 0:09^*^	4:19 ± 0:10^*^			4:13 ± 0:13^*^	
Best H	5:19 ± 0:01	4:50 ± 0:04	4:09 ± 0:09			4:01 ± 0:02	
(Range)	(5:18–6:04)	(4:47–5:12)	(4:02–4:33)			(4:00–4:30)	
SEG Lap	3:54 ± 0:03	3:34 ± 0:08^*^	3:10 ± 0:07^*^			3:01 ± 0:06^*^	
Best H	3:51 ± 0:01	3:34 ± 0:08	3:05 ± 0:01			2:56 ± 0:01	
(Range)	(3:51–4:03)	(3:25–3:42)	(2:58–3:28)			(2:55–3:09)	
BRH Lap	4:37 ± 0:17	4:01 ± 0:11^*^	3:14 ± 0:08^*^			3:10 ± 0:10^*^	
Best H	4:21 ± 0:04	3:48 ± 0:04	3:10 ± 0:11			3:02 ± 0:09	
(Range)	(4:18–5:08)	(3:43–4:18)	(3:03–3:30)			(2:56–3:22)	

Lap time (in min:sec ± SD) of Mixed H1–4 Team Relay and mean of best handbiker (Best H) per classe measured during Paracycling road UCI World Cup (Ro: Roma, May 2012 — 6 laps of 2.5 km and Seg: Segovia, June 2012 — 9 laps of 2 km) and Paralympic Games (BRH: Brands Hatch, September 2012 — 9 laps of 2 km).

* Significant different with MH1 time (P < 0.05).

δ Significant different with WH2 time (P < 0.05).

ϒ Significant different with WH3 time (P < 0.05).

These differences in TR performance could be explained in part by some different functional and physiological responses between tetraplegia and paraplegia. The measurement aggregation and weighting of the MH1 by manual muscle testing grade (Hislop and Montgomery, 2002) showed a limited elbow extension with a muscle score of grade 6 (total of both triceps), limited handgrip and no balance of the trunk. In their meta-analysis, Haisma et al. (2006) noted that the muscle strength of the upper extremity was comparable between paraplegic subjects and the age- and gender-matched able-bodied population. However, in subjects with cord injury at the cervical lesion, shoulder strength was reduced to 50% of normal predicted values. By isokinetic measurements, Bernard et al. (2004) reported an influence of the anatomical level of spinal lesion on shoulder strength and therefore wheelchair propulsion: lesser was the anatomical site of spinal injury, higher were the values of peak torque and mean power of external rotators. Tweedy and Vanlandewijck (2011) compared the tetraplegic and paraplegic performance on athletic track distances ranged from 100 to 800 m. They showed an incidence of the impairment on the functional possibilities and the gestural efficiency. Although the difference between arm cranking and pushing to propel hand-bike or athletic wheelchair, it is easy to notice the WH2 advantage whom used the entire upper limb and trunk musculature than MH1 who worked with their residual shoulder strength in arm cranking exercise. This difference could be more marked during crossing of stiff slope where in equal speed of movement, the strength developed on cranks could not be any more compensated with an increase of the cranking frequency and a gear ratio reduction.

In tetraplegia, respiratory function was also reduced. Numerous works suggested that the level of lesion is inversely correlated with respiratory function (Winslow and Rozovsky, 2003; Schilero et al., 2005) and the association between a reduced baseline airway caliber and a heightened vagomotor airway tone (Schilero et al., 2005). Haisma et al. (2006) in their critical review of 38 studies showed that the weighted mean for peak oxygen uptake and peak power output in tetraplegia subjects was reduced to 55–59% compared to paraplegia subject engaged in arm-cranking or hand-cycling exercise. Tetraplegia athletes are deprived of supraspinal sympathoadrenal control and the sympathic decentralization precludes cardio acceleration by lower peak of heart rate value ranged from 110 to 130 beats.min^−1^ during a maximal exercise (Schmid et al., 1998; Bhambhani et al., 2010). Compared to paraplegic, cardiac vagal withdrawal in tetraplegic subjects is not sufficient for full expression of cardiac acceleration during dynamic exercise (Coutts et al., 1983). Hence, Beekman et al. (1999) showed a lower speed and a lesser distance performed by tetraplegic subjects with a higher oxygen cost compared to subjects with paraplegia.

All these impairments finally places the MH1 in an unfavorable position compared to WH2 in TR constitution. However, the race topography may be impact the infraclass performance difference and so TR successful or unsuccessful. Uphill cycling field may be accentuated the difference in cycling performance between MH1 and WH2. The comparison of TR lap per category showed a greater performance for WH2 than MH1 at Roma, Segovia and London but with a lesser mean time difference during flat (Segovia, mean TR lap difference of 20 s between WH2 and MH1 winners) compared to uphill field events (mean TR lap difference of 35 s for London and 37 s for Roma between WH2 and MH1 win). The literature showed significant physiological and mechanical differences between tetraplegic and paraplegic athletes with spinal cord injury engaged in athletic wheelchair, arm-cranking and hand-cycling exercises. These are amplified during uphill field competition. This paper presents the difficulty to manage athletes with the same scoring point value but a different level of disability in the composition of a successful team relay. Team relay is the only one collective event allowing all H and all nations to participate in the international para-cycling competitions. With the current system of scoring, paraplegic women are favored with regard to the tetraplegic men to be a TR member. In one concerns to the tetraplegic athletes disappearance, we therefore recommend to UCI a high caution in the choice of the race topography.
